# Prenatal Bisphenol A Exposure Induces Preneoplastic Lesions in the Mammary Gland in Wistar Rats

**DOI:** 10.1289/ehp.9282

**Published:** 2006-08-29

**Authors:** Milena Durando, Laura Kass, Julio Piva, Carlos Sonnenschein, Ana M. Soto, Enrique H. Luque, Mónica Muñoz-de-Toro

**Affiliations:** 1 Laboratorio de Endocrinología y Tumores Hormonodependientes, School of Biochemistry and Biological Sciences, Universidad Nacional del Litoral, Santa Fe, Argentina; 2 Department of Anatomy and Cellular Biology, Tufts University School of Medicine, Boston, Massachusetts, USA

**Keywords:** bisphenol A (BPA), desmoplasia, endocrine disruptor, hyperplastic ducts, mammary tumor, mast cells, *N*-nitroso-*N*-methylurea (NMU)

## Abstract

**Background:**

Humans are routinely exposed to bisphenol A (BPA), an estrogenic compound that leaches from dental materials, food and beverage containers, and other consumer products. Prenatal exposure to BPA has produced long-lasting and profound effects on rodent hormone-dependent tissues that are manifested 1–6 months after the end of exposure.

**Objective:**

The aim of the present work was to examine whether *in utero* exposure to BPA alters mammary gland development and increases its susceptibility to the carcinogen *N*-nitroso-*N*-methylurea (NMU).

**Methods:**

Pregnant Wistar rats were exposed to BPA (25 μg/kg body weight per day) or to vehicle. Female offspring were sacrificed on postnatal day (PND) 30, 50, 110, or 180. On PND50 a group of rats received a single subcarcinogenic dose of NMU (25 mg/kg) and they were sacrificed on either PND110 or PND180.

**Results:**

At puberty, animals exposed prenatally to BPA showed an increased proliferation/apoptosis ratio in both the epithelial and stromal compartments. During adulthood (PND110 and PND180), BPA-exposed animals showed an increased number of hyperplastic ducts and augmented stromal nuclear density. Moreover, the stroma associated with hyperplastic ducts showed signs of desmoplasia and contained an increased number of mast cells, suggesting a heightened risk of neoplastic transformation. Administration of a subcarcinogenic dose of NMU to animals exposed prenatally to BPA increased the percentage of hyperplastic ducts and induced the development of neoplastic lesions.

**Conclusions:**

Our results demonstrate that the prenatal exposure to low doses of BPA perturbs mammary gland histoarchitecture and increases the carcinogenic susceptibility to a chemical challenge administered 50 days after the end of BPA exposure.

Epidemiologic studies and animal experimentation have revealed that alterations in the nutritional status of a developing fetus may predispose individuals to hypertension and coronary heart disease that become apparent in adulthood (Sallout and Walker 2003). Epidemiologic studies also suggest that the intrauterine hormonal milieu may predispose an individual to carcinogenesis. For example, increased risk of breast cancer correlated with twin dizygotic birth, a marker of high estrogen exposure (Braun et al. 1995), and preeclampsia, a marker of low estrogen exposure, was associated with lowered risk (Innes and Byers 1999). Currently, the concern about effects of prenatal estrogen exposure is focused on the exposure to environmental estrogens, which may affect mammary gland development and/or enhance the risk of breast cancer later in life.

Over the last 60 years, a plethora of synthetic hormonally active chemicals have been released into the environment. Meanwhile, an increase in endocrine-related diseases of the male reproductive system (Sharpe and Skakkebaek 1993) and testicular (Skakkebaek et al. 1998) and breast cancers (Davis et al. 1993; Sasco et al. 2003) have been reported.

Among these endocrine disruptors, bisphenol A (BPA) is receiving increased attention because of its high potential for human exposure. In fact, in a recent study, Calafat et al. (2005) reported the presence of BPA in 95% of urine samples. BPA has also been measured in human sera (mean ± SE: adult men, 1.49 ± 0.11 ng/mL; adult women, 0.64 ± 0.10 ng/mL) (Takeuchi and Tsutsumi 2002), and in human maternal and fetal plasma and in placental tissue at birth (Ikezuki et al. 2002; Schonfelder et al. 2002). BPA is used in the preparation of epoxy resins and in the manufacture of polycarbonate plastics and other consumer products (Krishnan et al. 1993; Steiner et al. 1992). BPA has been found in foods (4–23 μg/can), beverages (7–58 μg/g), and saliva (90–913 μg/saliva collected in a 1-hr period after application of dental sealant) in concentrations that were sufficient to induce the proliferation of estrogen target cells in culture (Biles et al. 1999; Brotons et al. 1995; Olea et al. 1996). Recognizing that it is not feasible to generate accurate exposure levels from the existing data, we have chosen to administer 25 μg BPA/kg body weight(bw)/day, which falls just 2.5-fold above the estimated daily intake of 0.01 mg/kg/day set by the European Commission (EC 2002).

In rodents, BPA has been shown to traverse the placenta (Takahashi and Oishi 2000), and it is also present in follicular fluid, amniotic fluid, and fetal serum during pregnancy (Zalko et al. 2003). The developing embryo is particularly susceptible to chemicals in general and hormones in particular (Bern 1992). As put succinctly by Gilbert (1997), “the construction of an organ can be affected by chemicals that have no deleterious effects on the normal functioning of that organ.” Previously, we demonstrated that perinatal exposure to BPA has profound effects on rodent hormone-dependent tissues long after exposure elapsed (Markey et al. 2001; Muñoz-de-Toro et al. 2005; Ramos et al. 2001, 2003). In the mammary gland, BPA altered development at the biochemical, cellular, and tissue levels of organization. Of particular interest were the increase in the number of terminal end buds and terminal ends (because these are thought to be the sites where carcinomas originate) and the increase in ductal density and sensitivity to estradiol, which also suggests increased susceptibility to mammary cancer (Markey et al. 2001; Muñoz-de-Toro et al. 2005). Hence, we hypothesize that peri-natal exposure to low doses of BPA increases the risk of developing mammary cancer.

The rat mammary carcinogenesis model is one of the most widely used surrogate models because it closely mimics the human disease allowing elucidation of the influence of host factors, both on the initiation of the neoplastic process and on the susceptibility according to age and reproductive history. In rats, tumors can be chemically induced by the administration of either dimethylbenz[*a*]anthracene (Russo and Russo 1996) or *N*-nitroso-*N*-methylurea (NMU; Thompson and Adlakha 1991). The sensitivity of the mammary gland to neoplastic transformation depends on the stage of mammary gland differentiation at the time of carcinogenic stimuli (Russo and Russo 1996). NMU can also induce mammary carcinomas in parous rats (Yang et al. 1999), although at a lower incidence than in sexually immature (Thompson et al. 1995) and peri-pubertal rats (Maffini et al. 2004; Thompson and Adlakha 1991).

Therefore, the aim of the present work was to extend our investigation on the susceptibility of mammary tissue to NMU-induced neoplasia following *in utero* exposure to BPA.

## Materials and Methods

### Animals

We used sexually mature female rats of the Wistar-derived strain bred at the Department of Human Physiology (School of Biochemistry and Biological Sciences, Santa Fe, Argentina). Animals were maintained in a controlled environment (22 ± 2°C; 14 hr of light from 0600 to 2000 hours) and had free access to pellet laboratory chow (Cooperación, Buenos Aires, Argentina). The concentration of phytoestrogens in the diet was not evaluated; however, because feed intake was equivalent for control and BPA-treated rats (unpublished observations) we assumed that animals in the experimental and control groups were exposed to the same levels of phytoestrogens. To minimize other exposure to endocrine-disrupting chemicals, rats were housed in stainless steel cages and tap water was supplied *ad libitum* in glass bottles with rubber stoppers. Animals were treated humanely and with regard for alleviation of suffering in accordance with the principles and procedures outlined in the *Guide for the Care and Use of Laboratory Animals* (Institute of Laboratory Animal Resources 1996).

### Experimental procedures

Females in proestrous were caged overnight with males of proven fertility. The day that sperm was found in the vagina was designated day 1 of pregnancy [gestation day 1 (GD1)]. Pregnant animals were assigned to one of two groups, with 11–14 mothers/group: dimethyl sulfoxide (DMSO; vehicle-treated control) or 25 μg BPA/kg bw/day (25 BPA). On GD8, corresponding to the beginning of organogenesis in the fetus, rats were weighed and implanted subcutaneously with a miniature osmotic pump (model 1002; Alza Corp., Palo Alto, CA, USA), which delivered 25 BPA (Sigma-Aldrich de Argentina S.A., Buenos Aires, Argentina) or DMSO (99.9% molecular biology grade; Sigma-Aldrich de Argentina S.A.). Both BPA and DMSO were released continuously for 14 days (from GD8 to GD23) at a rate of 0.25 μL/hr. Offspring were delivered on GD23 and weaned from their mothers at postnatal day 21 (PND21). We evaluated the effect of treatment in female offspring.

The first experiment was designed to study whether *in utero* BPA exposure affects the development of the rat mammary gland. These animals were sacrificed at prepuberty (PND30), after puberty (PND50), and adulthood (PND110 and PND180). Puberty onset was determined by examining vaginal opening, and body weight was recorded once each week during the study period.

The second experiment was designed to evaluate whether prenatal exposure to BPA enhanced the responsiveness of the mammary gland to chemically induced mammary pre-neoplastic/neoplastic lesions. To select a sub-carcinogenic dose of NMU, a pilot experiment was performed in which we tested the carcinogenic effect of 25 NMU (25 mg/kg bw; Sigma-Aldrich de Argentina S.A.), taking as reference the tumor incidence in rats receiving NMU (50 mg/kg bw), a dose previously defined as carcinogenic (Thompson et al. 1995). Fifty-day-old virgin rats received a single intra-peritoneal (ip) injection of either 25 NMU or 50 NMU (positive control) dissolved in 0.9% NaCl, acidified to pH 4.0 with acetic acid (Thompson and Adlakha 1991).

Once the subcarcinogenic NMU dose was established, female offspring from the DMSO group received either 25 NMU (control) or 50 NMU (positive control) in a single ip dose at PND50, and female offspring from the 25 BPA group received 25 NMU. The groups generated were as follows: DMSO + 25 NMU (*n* = 16), DMSO + 50 NMU (*n* = 10), and 25 BPA + 25 NMU (*n* = 21). Whereas all rats treated with 50 NMU were sacrificed at PND180, rats receiving 25 NMU were sacrificed either at PND110 or PND180. All animals were weighed and palpated biweekly for mammary tumor detection beginning 1 week after the NMU administration; however, to detect early nonpalpable lesions, we sacrificed a subset of 25 NMU rats and the corresponding controls on PND110. To evaluate NMU carcinogenic activity and rat strain susceptibility, we sacrificed animals injected with 50 NMU (positive control) on PND180.

### Tissue samples

Two hours before sacrifice, all rats were injected ip with bromodeoxyuridine (BrdU; 6 mg/100 g bw; Sigma-Aldrich de Argentina S.A.) to determine the proliferative index in the mammary gland stroma and epithelium. Abdominal–inguinal mammary gland chains were dissected out bilaterally. One chain was processed for whole mount (Thompson et al. 1995) and the other was fixed in 10% buffered formalin for 6 hr at room temperature and embedded in paraffin. In animals that had received NMU and were sacrificed on PND180 (DMSO + 25 NMU, DMSO + 50 NMU, and 25 BPA + 25 NMU), both mammary gland chains were whole mounted to facilitate the visualization of macroscopic and/or microscopic lesions. To localize microscopic lesions, the whole mounts were observed under a Leica stereomicroscope, (Leica Inc., Buffalo, NY, USA) and microscopic or macroscopic lesions were removed and embedded in paraffin for histologic analysis (Singh et al. 2000). Serial 5-μm paraffin sections were mounted on slides coated with 3-aminopropyl triethoxysilane (Sigma-Aldrich de Argentina S.A.) and stained with hematoxylin and eosin (H&E) or used for immunohistochemistry.

### Immunohistochemistry

For immunohistochemistry, sections were deparaffinized and dehydrated in graded ethanols. We evaluated BrdU incorporation by immunohistochemistry (BrdU antiserum; Novocastra Laboratories Ltd., Newcastle upon Tyne, UK) after acid hydrolysis for DNA denaturation and a microwave pretreatment for antigen retrieval (Kass et al. 2000). Sections used for immunostaining cytokeratin 8 (CK8; The Binding Site, Birmingham, UK) and p63 (Santa Cruz Biotechnology, Santa Cruz, CA, USA) were also subjected to a microwave pretreatment for antigen retrieval. Endogenous peroxidase activity and nonspecific binding sites were blocked. To identify mast cells, we used proteinase I (specifically, the rat mast cell proteinase I, RMCP-I; Moredun Scientific Ltd., Edinburgh, Scotland) immunostaining following the immunoperoxidase technique after periodic acid and sodium borohydrate treatment to block endogenous peroxidase activity (Varayoud et al. 2004). Primary antibodies were incubated overnight at 4°C at dilutions showed in Table 1. Reactions were developed by the avidin-biotin peroxidase method using diaminobenzidine (DAB) (Sigma-Aldrich de Argentina S.A.) as a chromogen substrate. Samples were counterstained with Harris’ hematoxylin (Biopur, Rosario, Argentina) for BrdU labeling or with Mayer’s hematoxylin for CK8, p63, and RMCP-I detection, and mounted with permanent mounting medium (PMyR, Buenos Aires, Argentina). Each immunohistochemical assay included positive and negative controls. Negative controls were incubated with nonimmune serum (Sigma-Aldrich de Argentina S.A.).

### In situ *detection of apoptosis*

The apoptotic cells were identified as previously described (Ramos et al. 2002) using the TUNEL technique (ApopTag, Serologicals Corporation, Norcross, GA, USA). Briefly, after incubation with proteinase K (5 μg/mL) (Intergen Co., Purchase, NY, USA) for 10 min at 37°C, sections were treated with hydrogen peroxide in phosphate-buffered saline for 10 min at room temperature to quench endogenous peroxidase activity. The incubation with a mixture containing digoxigenin deoxynucleotide triphosphate, unlabeled deoxynucleotide triphosphate, and terminal transferase enzyme was developed in a humidified chamber at 37°C for 1 hr. Subsequently, the reaction was visualized using anti-digoxigenin-peroxidase and DAB. Samples were counterstained with Mayer’s hematoxylin, and then dehydrated and mounted with PMyR. The negative control slides were prepared similarly, except that distilled water was added instead of terminal transferase enzyme. For a positive control, we processed an involuting mouse mammary gland collected 4 days after weaning in an identical manner.

### Morphometry and image analysis

#### Percentage of hyperplastic ducts

The percentage of hyperplastic ducts was quantified in H&E-stained sections. The primary criterion used for diagnosing hyperplasia was the presence of an increased number of epithelial layers within the ducts (Singh et al. 2000); only ducts with four or more layers of epithelial cells were considered hyperplastic. To obtain the percentage of hyperplastic ducts, we evaluated three mammary gland sections, at least 30 μm apart, and analyzed 50 ducts per section.

#### Stromal nuclei density

We recorded images of the fat pad using a Sony ExwaveHAD color video camera (Sony Electronics Inc., Park Ridge, NJ, USA) attached to an Olympus BH2 microscope (Olympus Optical Co., Ltd., Tokyo, Japan), which was prepared for Köhler illumination. The resolution of the images was set to 640 × 480 pixels and the final screen resolution was 0.103 μm/pixel. We evaluated two sections for each specimen and 40 representative fields in each section, covering a total area (*a_t_*) of 0.1 mm^2^. To quantify the fat pad area occupied by stromal nuclei (*a_n_*), we created an automated sequence operation using Auto-Pro macro language (Image Pro-Plus 4.1.0.1 system; Media Cybernetics, Silver Spring, MD, USA). Infiltrating cells and blood vessels cells were not included. The ratio between *a_n_* and *a_t_* was designated as the stromal nuclei density.

#### Evaluation of immunostained tissues

We evaluated immunostained tissue sections using an Olympus microscope with a Dplan 100 × objective (Olympus). Percentages of BrdU positives and apoptotic cells were determined separately in each cellular compartment (parenchyma and stroma) of the whole mammary section (at least 2,000 epithelial cells and 1,000 stromal cells per section). The ratio between BrdU positive cells and the apoptotic index (AI) was also calculated.

To obtain data regarding the number of mast cells in mammary tissue, we used a point counting procedure (Luque et al. 1996). We used a square grid inserted in a focusing eyepiece and determined the volume densities of mast cells. The volume density ratio was defined as the number of incident points in the studied cell (mast cell) divided by the total number of incident points in the volume unit (whole mammary gland).

We used immunostaining with CK8 (a marker for epithelial cells) and p63 (a marker for myoepithelial cells) to assess whether epithelial cells invaded the surrounding stroma.

### Statistics

Statistical analyses were performed using the Mann-Whitney *U* test; *p <* 0.05 was accepted as indicating a significant difference between groups.

## Results

### BPA exposure resulted in early puberty in female offspring

**Figure 1:**
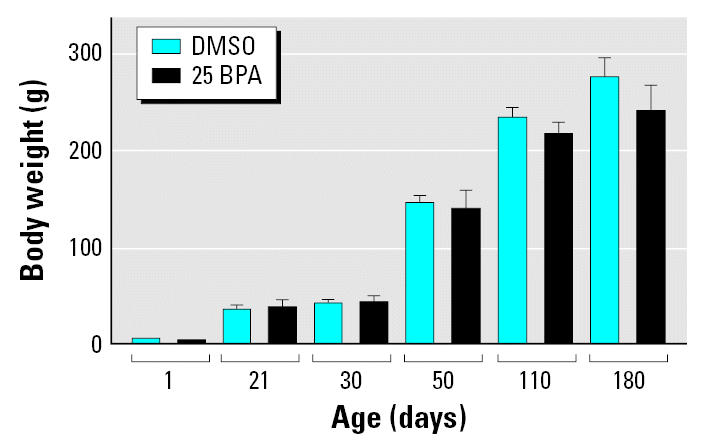
Body weight gain from birth to adulthood in female rats treated *in utero* with BPA. The female body weight was not modified by the BPA treatment during the evaluated period.

Female offspring exposed *in utero* to 25 BPA exhibited advanced puberty, measured as early vaginal opening compared to controls (Table 2). However, early vaginal opening was not associated with modifications in body weight. We found no significant difference in body weight between BPA-treated and control female offspring from birth to adulthood (PND180) (Figure 1).

### BPA-induced alterations of mammary gland organization in female offspring were evident after puberty

**Figure 2:**
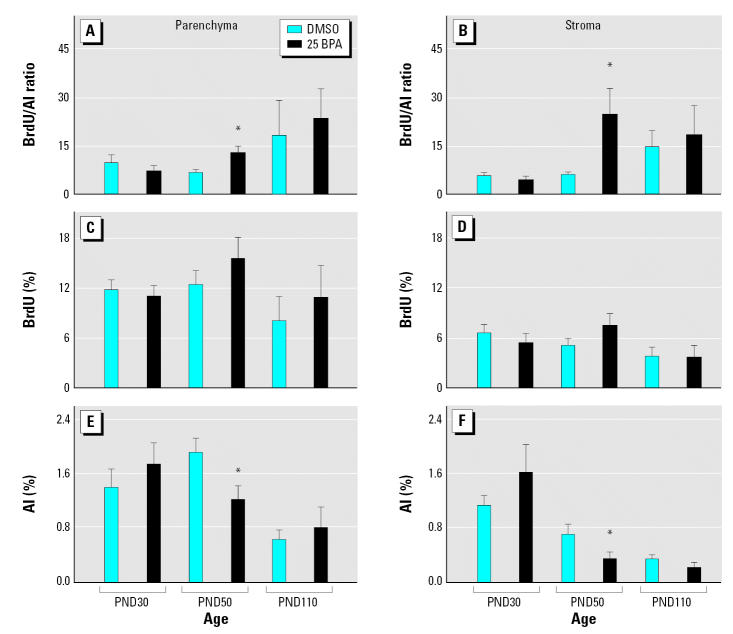
Mammary gland growth rate (BrdU/AI ratio; *A, B*), BrdU incorporation (*C, D*), and AI (*E, F*) quantified on PND30, PND50, and PND110 in mammary gland parenchyma and stroma of female offspring exposed *in utero* to BPA. Bars represent mean ± SE (at least six animals per group). *Statistically significant difference between BPA-treated animals and their respective controls for each PND (*p* < 0.05; Mann-Whitney *U* test).

**Figure 3:**
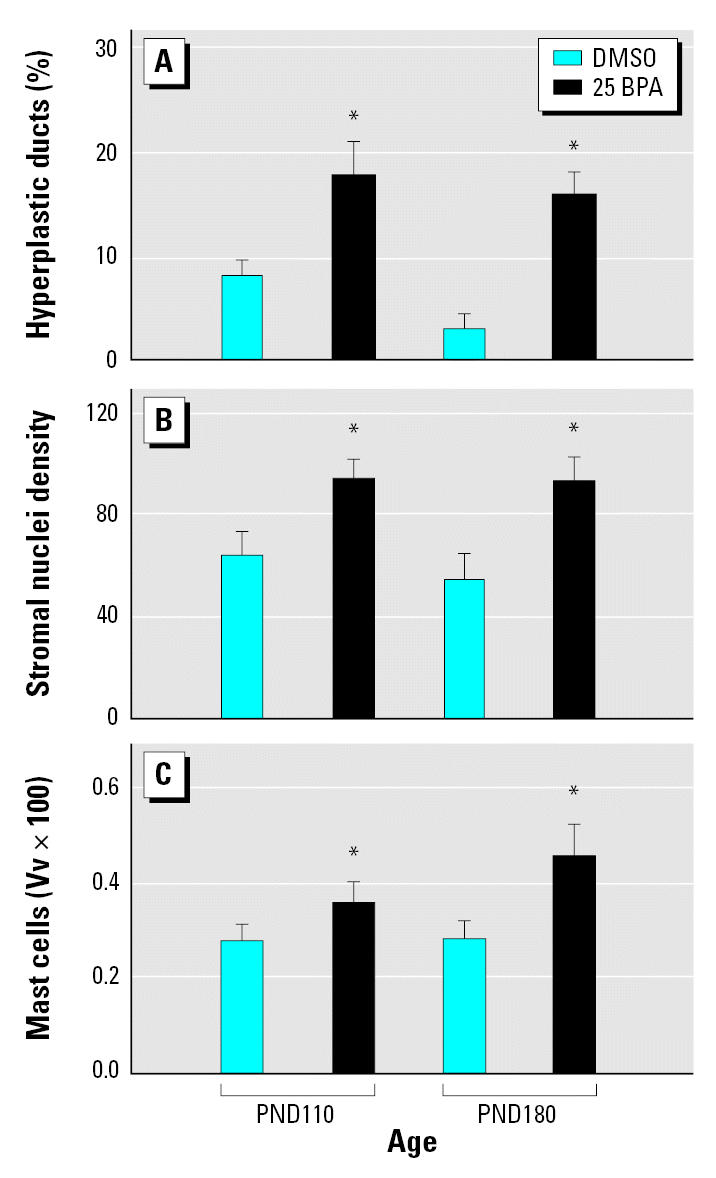
Quantitative evaluation of the histomorphologic changes in the mammary gland of female offspring treated *in utero* with BPA shown as the percentage of hyperplastic ducts (*A*), stromal nuclei density (*B*), and volume density (Vv) of mast cells (*C*). Bars represent mean ± SE (at least six animals per group). *Statistically significant difference between BPA-treated animals and controls (*p* < 0.05; Mann-Whitney *U* test).

**Figure 4:**
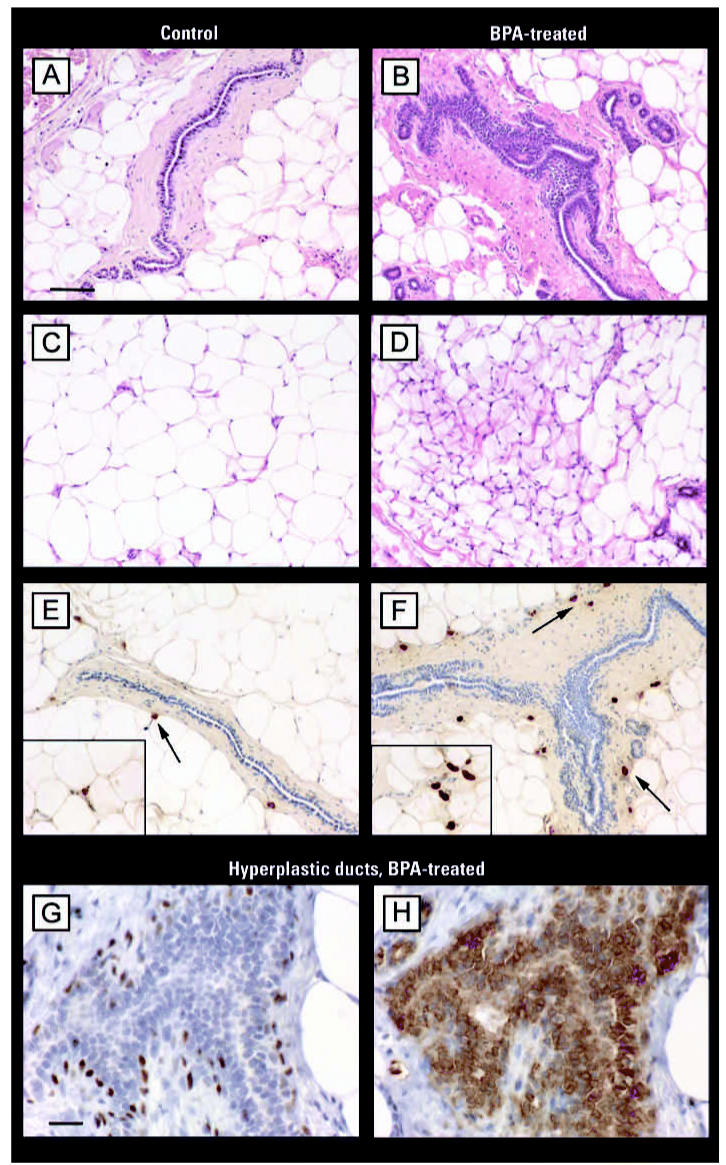
Representative photomicrographs of mammary glands from adult females (PND110) exposed *in utero* to vehicle (control; *A, C, E*) or 25 BPA (*B, D, F, G, H*). Tissue sections were either stained with H&E (*A–D*) or immunostained to identify mast cells (*E–F*), myoepithelial cells (*G*), or epithelial phenotype (*H*). Differences between normal ducts in control (*A*) and hyperplastic ducts (*B*) in BPA-treated animals are shown. The adipose tissue of the control mammary gland (*C*) consists of mainly fat cells, with few fibroblasts or blood vessels. Treatment with 25 BPA (*D*) promoted a significant increase of nuclear density in the stromal compartment. After BPA treatment, we found an increase in the volume density of mast cells (arrows) surrounding the hyperplastic duct (*F*) compared with few mast cells observed near the normal duct (*E*). The insets in (*E*) and (*F*) show mast cells at higher magnification. (*G*) and (*H*) show a higher magnification of a hyperplastic duct from a BPA-treated mammary gland; the epithelial phenotype of the cells layers within the hyperplastic ducts was confirmed by the positive CK8 immunostaining (*H*), whereas myoepithelial cells were labelled with p63 (*G*). Bars = 75 μm.

After puberty, at PND50, both the mammary gland parenchyma and stroma of animals prenatally exposed to BPA exhibited a higher BrdU/AI ratio (Figure 2A, B). This alteration in cellular turnover was mainly due to an inhibition of apoptosis; significantly lower AIs were observed (Figure 2E, F) while proliferative indices were slightly increased (Figure 2C, D). Changes in mammary tissue growth rate (Figure 2A–F) and the morphologic features of the stroma became apparent only after puberty. At PND30, we found no differences between the mammary glands of BPA- and vehicle-treated rats. At PND110 and PND180, we observed a significant increase in the percentage of hyperplastic ducts in BPA-treated animals relative to vehicle-treated controls (Figure 3A). Even though structures resembling hyperplastic ducts were identified in control groups, they represented < 10% of evaluated ducts. At PND110 and PND180, an increase in both the stromal nuclear density and the number of mast cells surrounding the hyperplastic ducts was found (Figure 3B, C; for a detailed view, see Figure 4E, F).

The mammary gland stroma of BPA-treated animals also exhibited morphologic changes in the extracellular matrix. A dense stroma layer was formed around mammary epithelial structures, and a fibroblastic stroma replaced the normal adipose tissue of the mammary gland exhibited by controls. The presence of such fibroblastic-like stroma, which also includes inflammatory cells, indicates a desmoplastic reaction (Figure 4A–D).

### Prenatal BPA exposure enhanced NMU effects on rat mammary glands

Results from the pilot experiment indicated that mammary tumor incidence after NMU administration at 180 days of age (almost 19 weeks after chemical carcinogen injection) in Wistar rats were 0% (0/10) for the group receiving 25 NMU and 83.3% (5/6) for 50 NMU. Thus, 25 NMU was considered a subcarcinogenic dose and was used to test our hypothesis.

Females treated *in utero* with vehicle (DMSO) and later with the subcarcinogenic NMU dose (25 mg/kg) showed no change in the number of hyperplastic ducts at PND110, whereas at PND180 a significant increase was found (Table 3). Moreover, in rats treated *in utero* with 25 BPA, the subcarcinogenic NMU dose (25 mg/kg) induced a significant increase in hyperplastic lesions at PND180 (Table 3). The differences between both treatments were statistically significant at PND180, indicating a positive interaction suggestive of an additive effect (Table 3).

**Figure 5:**
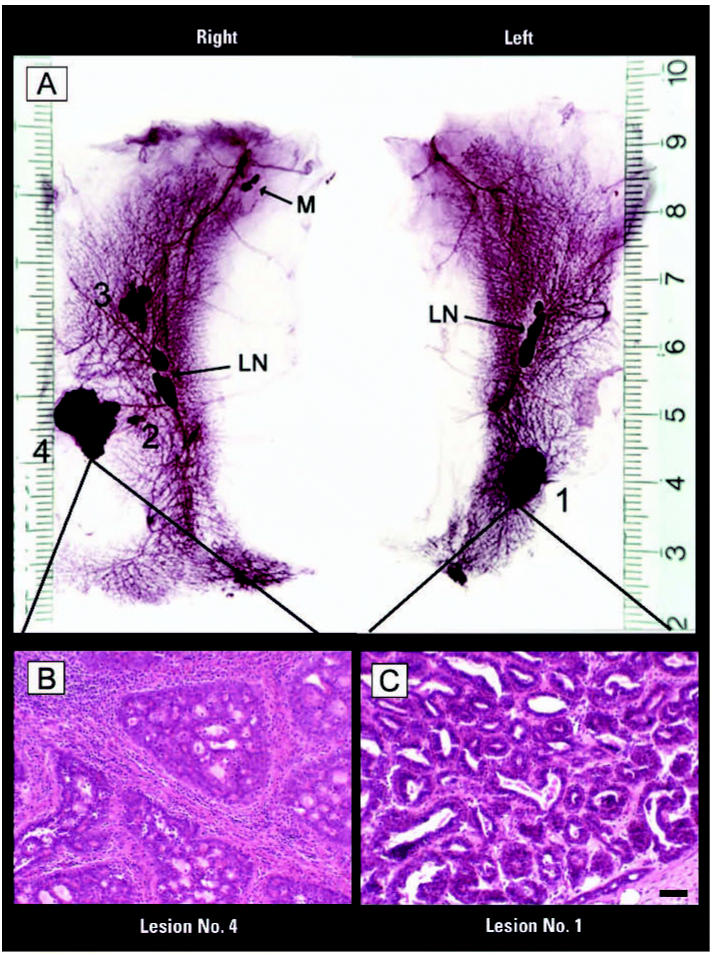
Whole mounts (*A*) and histologic sections of the abdominal inguinal mammary gland chains (*B, C*) from female rats treated *in utero* with 25 BPA and exposed after puberty to the subcarcinogenic dose of NMU. Abbreviations: LN, lymph nodes; M, muscle. Whole mounts (*A*) show gross lesions. H&E-stained lesions are classified as ductal carcinoma *in situ* of the cribriform (*B*) and mixed (*C*; cribriform and papillar) types. Bar = 70 μm (*B, C*).

In addition to increasing the incidence of preneoplastic lesions, *in utero* exposure to 25 BPA enhanced the response to the sub-carcinogenic NMU dose: at PND180, 13.3% (2/15) of animals developed mammary malignancies. All tumors were encapsulated and of solid consistency, and the stromal response demonstrated by fibrosis and mononuclear infiltration (mainly lymphocytes and eosinophils) was a common feature. CK8 immunostaining patterns ruled out stromal invasion by epithelial cells. Tumors were classified as ductal carcinoma *in situ* with cribriform (Figure 5B), papillary, or mixed pattern (cribriform and papillary) (Figure 5C). Other than neoplastic mammary lesions, we observed a salivary gland neoplasia and a cytosteatonecrosis (a large droplet of lipid surrounded by connective tissue with abundant eosinophilic infiltration) in the animals treated with a subcarcinogenic dose of NMU. Mammary tumors in rats treated with the carcinogenic dose of NMU (positive control) reached an incidence of 70% (7 of 10) and were classified as invasive adenocarcinoma of papillary, cribriform, or mixed pattern. Two malignant thyroid gland tumors of follicular origin were also diagnosed. Results regarding incidence of tumors and/or hyperplastic lesions and tumor multiplicity are summarized in Table 3.

## Discussion

In the present study we examined the influence of prenatal BPA exposure on the post-natal development of the female mammary gland and on susceptibility to NMU-induced mammary neoplasia. Prenatal exposure to BPA resulted in an increased number of pre-neoplastic lesions, namely, ductal hyperplasias involving the epithelial compartment and stromal alterations in the vicinity of the affected ducts. Because these effects were not apparent before puberty, it is plausible to infer that mammary glands of BPA-exposed rats may be more sensitive to estrogen than the mammary glands of unexposed animals. In fact, increased responses to estradiol were reported in the mammary glands of mice exposed perinatally to BPA (Muñoz-de-Toro et al. 2005).

Carcinogenesis is a complex process in which interactions between stromal and epithelial cells play an important role (Barcellos-Hoff 2001; Maffini et al. 2004, 2005). Moreover, a recurrent concept in cancer biology is that neoplastic transformation represents development gone awry. From this perspective, it is reasonable to hypothesize that extemporaneous exposure to estrogens or xenoestrogens during fetal development may alter the reciprocal interactions that induce and maintain tissue organization, and that these alterations in turn generate abnormal tissue structures and altered control of cell proliferation. Thus, a marked stromal reaction and a deregulation of growth rate in both the parenchyma and the stroma (Chrenek et al. 2001; Noël and Foidart 1998) would be observed even at early stages of neoplastic transformation. At PND50 in the BPA-exposed group (i.e., immediately after puberty), we observed significant deregulation of mammary gland growth as a consequence of two trends: an increase in proliferation and a decrease in apoptosis. Alterations in these two processes modified cellular turnover, a phenomenon observed during early stages of mammary carcinogenesis (Shilkaitis et al. 2000). The present results support our previous finding that prenatal exposure to BPA increases the sensitivity of the developing mammary gland to endogenous estrogen, thereby creating a permissive state that can lead to malignancy (Muñoz-de-Toro et al. 2005). In this context, we suggest that the increased incidence of hyperplastic ducts and increased stromal nuclear density observed in adult animals (PND180) may be a consequence of the cellular turnover deregulation that occurred earlier in life (i.e., around puberty). Hyperplastic ducts are considered premalignant structures and the precursors of neoplastic lesions (Singh et al. 2000).

The alterations observed in the mammary gland stroma of females exposed to BPA *in utero* may predispose to neoplastic development. In this regard, recent observations suggest that the desmoplastic reaction in breast cancer is the result of altered epithelial–stroma interactions and that accumulation of stromal fibroblasts provides both structural and hormonal support for the tumor tissue (Deb et al. 2004; Meng et al. 2001). Other structural features, such as an increase in matrix rigidity, may perturb tissue architecture, enhancing cell growth and tumor metastasis (Akiri et al. 2003; Paszek et al. 2005). Thus, the fibrotic response observed in the mammary glands of adult animals exposed prenatally to BPA may play a permissive, if not cocausal, role regarding NMU-induced carcinogenesis. In this context, it is relevant to recall that Maffini et al. (2004) observed that epithelial mammary tumors could be induced after recombination of unexposed normal epithelial cells with NMU-exposed stroma.

In BPA-exposed animals, we observed an increased number of mast cells in the mammary gland stroma that were spatially associated with hyperplastic ducts. Mast cells are multifunctional effector cells of the immune system that produce and release a wide variety of mediators. Mast cells have been implicated in promoting angiogenesis in reproductive tissue (Varayoud et al. 2004) and within tumors (Aoki et al. 2003), but their precise effects on tumor growth remains unclear. Dabbous et al. (1986, 1995) proposed that mast cells increase proliferation of tumor cells and facilitate tumor invasion by promoting collagenolytic activities. Furthermore, using a mast cell–stabilizing compound they observed inhibition of tumor growth (Dabbous et al. 1991). Indeed, mast cells have been linked to intraductal proliferations that could progress to carcinoma *in situ* and to invasive carcinoma (Russo and Russo 1996). The factors that regulate the progression of normalcy to preneoplasia and neoplasia are unknown; however, mast cell degranulation could contribute directly to this sequence by modifying stroma–epithelium interactions either by stimulating angiogenesis or through extracellular matrix degradation (Aoki et al. 2003; Dabbous et al. 1986, 1995; Folkman 1986) The increase of the mast cell number in the mammary gland of BPA-exposed animals also buttresses the notion of the permissive effect exerted by prenatal exposure to BPA on chemically induced carcinogenesis.

Several factors contribute to the induction of rat mammary tumors, among them, the age of animals at the time of chemical carcinogen exposure, the carcinogen itself, and the susceptibility of the rat strain. We considered each of these factors in our study. Although the highest incidence of tumors induced by NMU has been obtained by applying the carcinogen in animals at 21 days of age (Thompson et al. 1995), we decided to inject our animals at PND50 for two reasons: the 50- to 55-day period is one where maximal density of highly proliferating terminal end buds occurs (Russo and Russo 1996), and our first experiment signaled that this is the period in which BPA-exposed animals showed the highest BrdU/AI ratio in the mammary glands. Regarding the carcinogen, we selected NMU because *a*) it has a short half-life (< 30 min) and does not need to be metabolized to become active; *b*) NMU tumors are mainly estrogen dependent, like human breast carcinoma (Rose et al. 1980); and *c*) the induced carcinomas are usually aggressive and locally invasive (Thompson and Adlakha 1991). In addition, our model was developed using Wistar rats, which are considered to have medium sensitivity to NMU (Isaacs 1986).

We observed a significantly increased number of ductal hyperplasias at PND110 and PND180 in BPA-treated animals compared with DMSO-treated animals; this suggests that prenatal BPA exposure increased the sensitivity of the gland to develop preneoplastic lesions. The administration of subcarcinogenic doses of NMU to animals exposed prenatally to vehicle produced no observable effects until PND180. At PND180, BPA-exposed animals that were treated with NMU exhibited a significantly higher number of ductal hyperplasias compared to animals that were not exposed to BPA; this suggests that prenatal BPA exposure increased the susceptibility of the gland to develop pre-neoplastic lesions as a response to NMU exposure. In addition, treatment with the subcarcinogenic dose of NMU (25 mg/kg/day) only induced carcinomas in the mammary glands of animals exposed prenatally to BPA.

In summary, we conclude that prenatal exposure to low, environmentally relevant doses of BPA may increase the risk of developing rat mammary tumors. The results reported here indicate that *in utero* BPA exposure *a*) induced alterations in the mammary gland at cellular and tissue levels that could be considered as preneoplastic lesions, and *b*) increased the susceptibility to the chemical carcinogen NMU, which resulted in the development of carcinomas. It is relevant to ask what is the significance of the results reported herein using a widely accepted surrogate model of breast carcinogenesis to that experienced in the human condition. Our observations strengthen arguments linking the recently reported increased incidence of endocrine-dependent human tumors, including those in the breast, to *in utero* exposure to minimal doses of xenoestrogens such as BPA, to which pregnant women are exposed.

## Figures and Tables

**Table 1:** Antibodies used for immunohistochemistry.

Antibody	Animal source	Clone	Concentration
BrdU	Mouse	85–2C8	1:100
RMCP-I	Rabbit		1:200
CK8	Sheep		1:400
p63	Mouse	4-A4	1:100

**Table 2:** Pregnancy-, nursing-, and fertility-related variables in rats treated with BPA.

Variable	DMSO	25 BPA
Percent successful pregnancies in pregnant dams	100 (*n =* 11)	100 (*n =* 14)
Mother’s weight gain during pregnancy [g (mean ± SD)]	112 ± 4	116 ± 15
Length of pregnancy (days)	23	23
No. of pups/litter (mean ± SD)	11 ± 3	9 ± 3
Percent females per litter (mean ± SD)	49.0 ± 17.1	42.6 ± 22.6
AGD of female offspring [mm (mean ± SD)]		
PND1	2.5 ± 0.5	1.8 ± 0.1
PND5	3.3 ± 0.6	3.0 ± 0.2
Age at vaginal opening [days (mean ± SD)]	39 ± 3	34 ± 1*

AGD, anogenital distance.

* Significantly different from control at *p* < 0.05 (Mann-Whitney *U* test).

**Table 3:** Effect of prenatal exposure to BPA and postnatal exposure to NMU on the incidence of premalignant and malignant lesions in the rat mammary gland.

Treatment			Results		
*In utero*	NMU (mg/kg)^a^	Day of sacrifice	Hyperplastic ducts (%)^b^	Tumor incidence	Tumor multiplicity^c^
DMSO	—	PND110	8.2 ± 1.5^c^	0/6	—
25 BPA	—	PND110	18.0 ± 3.2^e^	0/5	—
DMSO	25	PND110	5.3 ± 1.3^d^	0/9	—
25 BPA	25	PND110	14.8 ± 2.8^e^	0/9	—
DMSO	—	PND180	3.2 ± 1.3^f^	0/6	—
25 BPA	—	PND180	16.2 ± 2.3^e^	0/6	—
DMSO	25	PND180	15.7 ± 1.2^e^	0/10	—
25 BPA	25	PND180	35.5 ± 3.7^g^	2/15	2.5 ± 2.1
DMSO	50	PND180	19.5 ± 2.2^e^	7/10	1.5 ± 0.8

a Administered at PND50.

b Mean ± SE.

c Number of mammary tumors per rat (mean ± SE).

d–g Different letters denote statistical differences between groups (*p* < 0.05; Mann-Whitney *U* test).
